# An effective virus-based gene silencing method for functional genomics studies in common bean

**DOI:** 10.1186/1746-4811-7-16

**Published:** 2011-06-13

**Authors:** Claudia Díaz-Camino, Padmanaban Annamalai, Federico Sanchez, Aardra Kachroo, Said A Ghabrial

**Affiliations:** 1 Departamento de Biología Molecular de Plantas, Instituto de Biotecnología, Universidad Nacional Autónoma de México, Av. Universidad 2001, Colonia Chamilpa, CP 62210, Cuernavaca, Morelos, México; 2 Department of Plant Pathology, 201F Plant Science Building, University of Kentucky, 1405 Veterans Drive, Lexington, KY 40546-0312, USA

## Abstract

**Background:**

Common bean (*Phaseolus vulgaris *L.) is a crop of economic and nutritious importance in many parts of the world. The lack of genomic resources have impeded the advancement of common bean genomics and thereby crop improvement. Although concerted efforts from the "Phaseomics" consortium have resulted in the development of several genomic resources, functional studies have continued to lag due to the recalcitrance of this crop for genetic transformation.

**Results:**

Here we describe the use of a bean pod mottle virus (BPMV)-based vector for silencing of endogenous genes in common bean as well as for protein expression. This BPMV-based vector was originally developed for use in soybean. It has been successfully employed for both protein expression and gene silencing in this species. We tested this vector for applications in common bean by targeting common bean genes encoding nodulin 22 and stearoyl-acyl carrier protein desaturase for silencing. Our results indicate that the BPMV vector can indeed be employed for reverse genetics studies of diverse biological processes in common bean. We also used the BPMV-based vector for expressing the green fluorescent protein (GFP) in common bean and demonstrate stable GFP expression in all common bean tissues where BPMV was detected.

**Conclusions:**

The availability of this vector is an important advance for the common bean research community not only because it provides a rapid means for functional studies in common bean, but also because it does so without generating genetically modified plants. Here we describe the detailed methodology and provide essential guidelines for the use of this vector for both gene silencing and protein expression in common bean. The entire VIGS procedure can be completed in 4-5 weeks.

## Background

Common bean (*Phaseolus vulgaris*) is the world's most important food legume for direct human consumption in developing countries such as Latin America and Eastern Africa. Although common bean production occurs in a wide range of cropping systems and environments, Latin America with its 8 million hectares in common bean production is the most important region as it accounts for nearly half of the global output [1].

Thus far, common bean crop improvement through biotechnological approaches has been limited due to its recalcitrance for genetic transformation. Genetic transformation of common bean has been achieved by using biolistic or *Agrobacterium tumefaciens*-based methods, but with low efficiency [2-8] and thus is not amenable for high throughput molecular and genetic techniques for analysis of gene function. An efficient common bean root transformation protocol for different cultivars and landraces of *Phaseolus spp*. with *A. rhizogenes *K599 was recently reported [9]. This method, even though is useful to study genes involved in microbial symbiosis or root architecture, is not applicable to genes involved in other traits, such as those controlling shoot development and flowering.

Virus-induced gene silencing (VIGS) is an appealing reverse-genetic strategy that allows gene functional analysis in species not amenable to stable genetic transformation. However, its success relies on the ability of viral vectors to infect the plant species of interest. There are few examples of viral vectors that are suitable for use as VIGS vectors for legumes; these include pea early browning virus (PEBV), bean pod mottle virus (BPMV), a pseudorecombinant strain of cucumber mosaic virus (CMV) and apple latent spherical virus (ALSV). PEBV (genus *Tobravirus*) was successfully used in *Pissum sativum *[10], *Medicago truncatula *and *Lathyrus od*orata [11]. BPMV (genus *Comovirus*) has been widely used to silence genes or to express foreign proteins in *Glycine max *[12-16]. Vectors based on a pseudorecombinant strain of CMV (genus *Cucumovirus*) and ALSV (genus *Cheravirus*) have been used for VIGS in soybean [17-19].

**Figure 1:**
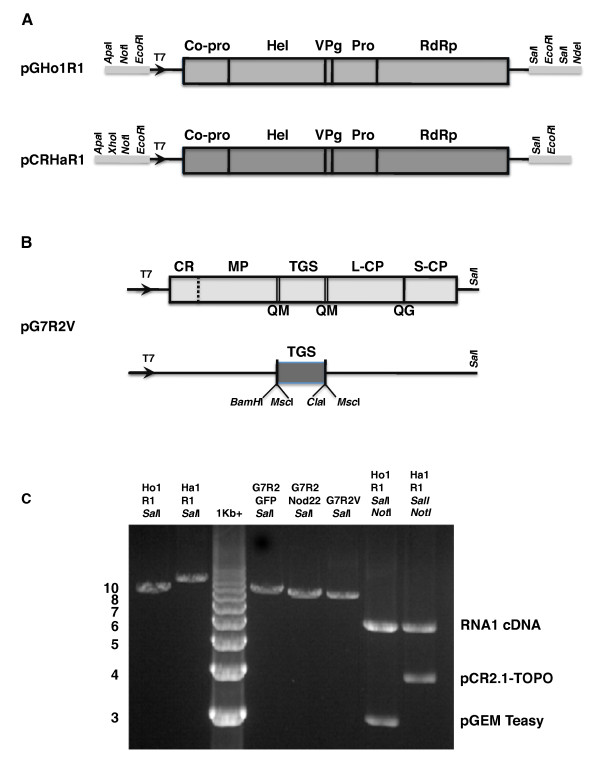
**Schematic representation of full-length cDNA clones of BPMV RNA1 and BPMV RNA2 vector (pG7R2V)**. (A) Schematic representation of BPMV RNA1 cDNA constructs used for generation of infectious transcripts. Plasmids pGHopR1 and pCRHanR1 contain full-length RNA1 cDNA of two strains of BPMV (K-Hop and K-Han, respectively) inserted downstream of a modified T7 promoter and cloned in pGEM Teasy and pCR2.1 TOPO, respectively [23]. Vertical lines indicate cleavage sites in the polyproteins; the designations of the mature proteins (protease co-factor, Co-pro; helicase, Hel; genome-linked protein, VPg; protease, Pro; and RNA-dependent RNA polymerase RdRp) are written above the relevant coding regions. For transcription, pGHopR1 and pCRHanR1 are linerized with *Sal*I (lanes HopR1*Sal*I and HanR1*Sal*I, respectively, Figure 1C) or, preferably for better yield, with *Sal*I and *Not*I (see corresponding lanes, first two lanes on the right, Figure 1C). (B) Genome organization of BPMV RNA2 and vector construction strategy. BPMV RNA2 is translated into two overlapping carboxy coterminal polyproteins. CR, RNA2 replication cofactor; MP, movement protein; L-CP, large coat protein; and S-CP, small coat protein. The vector pG7R2V [12] contains a GFP fragment (ΔGFP) inserted between the coding regions of MP and L-CP and also contains additional restriction sites (*Bam*HI and *Msc*I) for cloning of target gene sequences (TGS). A target gene can be cloned as a *Bam*HI-*Msc*I fragment in *Bam*HI-*Msc*I digested pGG7R2V vector. Alternatively, the target gene can be blunt-end ligated into *Msc*I-digested pGG7R2V. In constructing the vector, the Q/M cleavage site sequence between MP and L-CP (the dipeptide QM plus flanking sequences) was duplicated. A T7 RNA polymerase promoter sequence was engineered upstream the modified full length RNA2 cDNA and cloned into plasmid pGEM T easy to generate pG7R2V. The plasmid pG7R2V can be linearized by digestion with *Sal*I prior to transcription (lane G7R2V*sal*I; Figure 1C). (C). Restriction digestion of recombinant plasmids containing RNA1 cDNA (HopR1 and HanR1) and recombinant RNA2 (G7R2GFP, G7R2*Nod22 *and G7R2V) with *Sal*I or *Sal*I and *Not*I restriction enzymes and visualized after electrophoresis on 1.0% agarose gel and staining with ethidium bromide.

The BPMV genome is expressed via the synthesis and subsequent proteolytic processing of polyprotein precursors. RNA1 encodes five mature proteins required for replication (Figure 1A), whereas RNA2 encodes a putative cell-to-cell movement protein (MP) and two coat proteins (L-CP and S-CP). For developing BPMV-RNA2 as a vector, the target gene sequences were inserted into the BPMV vector recognition sites for *BamH*I and *Msc*I, which were engineered into the RNA2 within the coding sequences of MP and L-CP. Proteinase cleavage sites were created by duplicating the cleavage site between MP and L-CP to flank the heterologous sequences (Figure 1B). This ensures proper processing of the recombinant viral RNA2-encoded polyprotein (for details see reference #12 and legend for Figure 1).

As far as we know all commercially available cultivars of soybean are susceptible to BPMV infection. In contrast, only a few common bean varieties including the cultivar Black Valentine are susceptible to BPMV. To demonstrate gene silencing mediated by BPMV vectors in common bean, we targeted the genes (*PvNod22 *and *PvSACPDs*) that encode, respectively, nodulin 22 (Nod22) and stearoyl-acyl carrier protein desaturases (SACPDs) in the cultivar Black Valentine. Furthermore, we used the BPMV vector for expressing the green fluorescent protein (GFP) in common bean and shown stable GFP expression in all common bean tissues where BPMV was detected thus establishing that the BPMV vector is also suitable for expression of heterologous proteins in common bean.

## Methods

### Construction of viral vectors and in vitro transcription

For generating a *Nod22 *silencing vector, a 360-bp DNA fragment was amplified from a *Nod22 *cDNA clone [20] using sequence-specific primers with *Bam*HI and *Msc*I sites (printed in bold): *Nod22*-FWD-5'GAGGCG**GGATCC**CAGGCGCTGT-TG'3; *Nod22*-REV-5' GTCTTC**TGGCCA**CTCTCCGTGCCC3'. The GFP gene was amplified by PCR from plasmid pSITE-2CA [21] using the primer pair GFP-FWD 5'ATCCGGATCCGTGAGCAAGGGCGAGGAGCTGTT3' and *GFP*-REV-5' ACCTTGGCCACTTGTACAGCTCGTCCATGCCGAGAG3'. PCR amplification was performed at 94°C for 1 min, 55°C for 1 min and 72°C for 1 min for 30 cycles. The *GFP *and *Nod22 *amplified PCR products were gel purified and digested with *Bam*HI and *Msc*I and sub-cloned into pGG7R2-V previously digested with the same restriction enzymes and further verified by sequencing [12]. Construction of BPMV-VIGS for silencing *GmSACPDs *was previously described [13]. The recombinant BPMV-*SACPD *RNA2 transcript plus RNA1 from BPMV Kentucky strain Hopkins (K-Hop) or Hancock (K-Han) strains, were used for inoculation. To use plasmid pGG7R2-V as an empty vector control, the plasmid was first digested by *Msc*I and then re-ligated, giving rise to pGG7R2-M. This action removes the *GFP *fragment in pGG7R2-V [12]. Transcripts from pGG7R2-M, but not from pGG7R2-V, are infectious. Transcript from pGG7R2-M, plus transcript from RNA1 (subgroup I) cDNA from strain K-Hop or from RNA1 (subgroup II) cDNA from strain K-Han were used for inoculation. The wild type strain K-Hop, which induces very severe symptoms in soybean, is a naturally occurring partial diploid that is diploid for RNA 1 (subgroups I and II) and monoploid for RNA 2 (subgroup II; [22]). Monoploid RNA1 reassortants containing RNA1 (subgroup I) induces significantly less severe symptoms than the naturally occurring partial diploid strain K-Hop [22]. Plasmid constructs were used for in vitro transcription as previously described [23]. Briefly, capped RNA transcripts were synthesized by incubating 1 to 5 μg of linearized plasmids in a 100-μl reaction mixture containing 40 mM Tris-HCl, pH 7.5; 6 mM MgCl_2_; 2 mM spermidine; 10 mM dithiothreitol; 50 units of RNasin (Promega Corp., Madison, WI, U.S.A.); 0.5 mM each ATP, CTP, and UTP; 0.1 mM GTP; 0.5 mM cap-analogue (m^7^G[5γ]G) (New England Biolabs, Ipswich, MA, U.S.A.]; and 50 units of T7 RNA polymerase (New England Biolabs) at 37°C for 2 h. Yield and integrity of the transcripts were analyzed by electrophoresis on a 1.0% agarose gel. RNA1 transcript, derived from RNA1 cDNA, and recombinant RNA2 transcript were used to rub inoculate fully expanded unifoliate leaves of common bean or soybean.

### Plant inoculation and growth conditions

RNA transcripts were used to rub inoculate fully expanded unifoliate leaves of common bean (*Phaseolus vulgaris *cv Black Valentine) or soybean (*Glycine max *cv Essex). Mock, vector-inoculated, *PvNod22-, PvSACPD*-silenced or GFP expressing plants were analyzed 15 and 25 dpi. Four-six plants were included per treatment in each biological replicate. Plants were grown in a growth chamber at 24-26°C and 16 h photoperiod.

### Sample preparation, RNA extraction and soybean *Nod22 *RT-PCR analysis

Plant material from two biological replicates was collected 15 or 25 dpi, immediately frozen in liquid nitrogen and stored at -80°C. Total RNA from leaf and root tissues was isolated using the TRIzol reagent (Invitrogen) according to manufacturer's instructions. RNA in samples was then precipitated by adding lithium chloride (LiCl) to a 2M final concentration. Resultant pellets were re-suspended in water and the concentration and quality of each sample were determined using a NanoDrop ND-1000 spectrophotometer (NanoDrop Technologies) and by gel electrophoresis, respectively. All samples were subsequently treated with DNase I (Amp. grade, Invitrogen) to remove any residual DNA contamination. Reverse transcription and first-stranded cDNA synthesis was carried out using Superscript II (Invitrogen). Common bean and soybean RNA preparations were analyzed by RT-PCR using specific primers (Table 1). The number of amplification cycles was reduced from 30 to 26 for evaluating the relative differences in transcript levels.

**Table 1:** Sequences of primers used for RT-PCR and qRT-PCR.

Primer name	Primer sequence (5→3, forward, reverse)	Protein name
*nod22 forw*	ATGTTCCACAGTAGCAAAAACA	Nodulin 22
*nod22 rev*	TCACTGTACAAGCACAAGTCTC	Nodulin 22
*nod22*-ext.	GTCTTCGCCCGAAAAAGAAAGG GCAGAGGATTGCAACACCAAGA	Nodulin 22
*nod22*-int.	GAGACTGAGGGTATCGGCGACGTG GATTCTGGTAAACGGAACCTCCAC	Nodulin 22
*PvSACPD-C'*	TATGTACGACGGGGAAGACC GATTGGAGAAGCTGGAAGGT	Stearoyl-ACP desaturase (C-like)
*PvEf1-α*	GGTCATTGGTCATGTCGACTCTGG GCACCCAGGCATACTTGAATGACC	Elongation factor-1 alpha

### Primer design and qRT-PCR analysis

Gene-specific primers to generate 140-150 bp PCR products were designed using the OligoPerfect™ (Invitrogen) software. Gene specific primers to amplify *PvSACPD-C *like (*PvSACPD-C'*) and *PvEf1*-α were designed based on EST records available in the database (Table 1). Two pairs of non-overlapping *PvNod22 *specific primers were used to determine the real abundance of the *Nod22 *transcript (*nod22*-int. and *nod22*-ext.). By using a complex cDNA mixture, primers were preliminarily tested in semi-quantitative RT-PCR assays to verify the production of a unique PCR fragment of the expected length. Real-time RT-PCR reactions were performed in optical reaction tubes using an iCycler iQ5 apparatus (BioRad, Hercules, CA, USA). *Pvnod22 *and *PvSACPD-C' *transcript levels were determined with the SuperScript III SYBR Green One-Step qRT-PCR System (Invitrogen) according to the manufacturer's protocol in a final volume of 25 μL including 0.4 mM of each primer, 12.5 μL of the SYBR Premix (2X), 100 ng of total RNA, and sterile distilled DEPC-treated water. The cycling conditions were: 3 min at 50°C for cDNA synthesis, preheating for 5 min at 95°C followed by 40 cycles (denaturing for 15 s at 95°C, annealing and elongation for 15 s at 55.8°C and data acquisition at 81°C). A negative control reaction without template was also included for each primer combination. The melt curve protocol began immediately after amplification and consisted of 1 min at 55°C followed by 80-10 s steps with a 0.5°C increase in temperature at each step, to ensure the absence of primer-dimers and a single PCR product. Threshold values for threshold cycle (Ct) determination were generated automatically by the iCycler iQ5 software. Transcript levels for each of the target genes (*PvNod22 *and *PvSACPD-C'*) were normalized to the endogenous elongation factor *PvEf1*-α transcript level. Three technical replicates were analyzed for each biological replicate. Each biological replicate resulted in similar trends and we report a mean value for each experiment.

### Visualization of GFP expression in leaves and roots of HopV and HopGFP treated plants

GFP was observed under a Zeiss LSM 510 Meta confocal microscope attached to an Axiovert 200 M. GFP excitation was obtained at 488 nm using an Argon laser, an HFT UV 488/543/633 main dichroic excitation mirror and a BP 500-550 IR emission filter for detection. Simultaneously, autofluorescence was observed by exciting at 543 nm with a He/Ne laser; with the same main dichroic excitation mirror, a LP 560 emission filter and a BG 39 dichroic beam splitter. Images were processed using Adobe Photoshop 7.0 software (Adobe Systems Inc., Mountain View, CA, U.S.A.).

## Results

### BPMV-based VIGS vector effectively silences *Nod22 *expression in common bean

**Figure 2:**
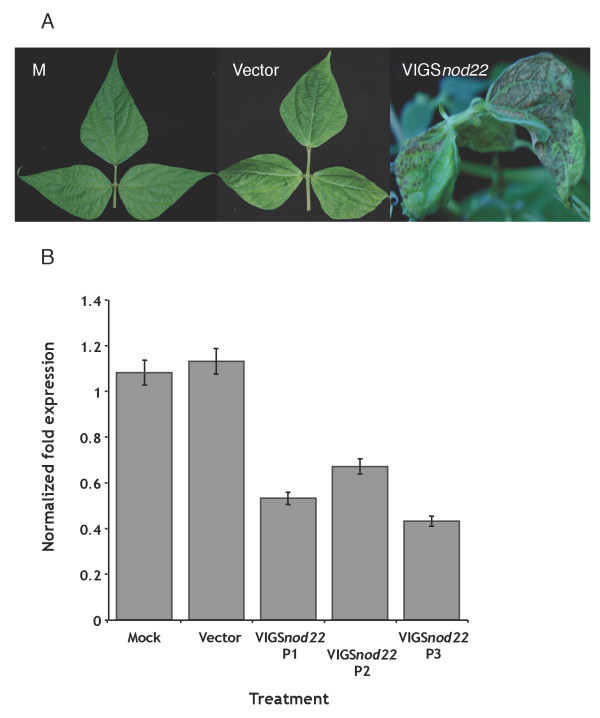
**Virus-induced gene silencing of *PvNod22 *in common bean plants mediated by the BPMV-RNA2 vector**. Silencing of *PvNod22 *induces necrotic phenotypes in 3 week-old common bean plants (upper panel, VIGS*Nod22*). Relative levels of expression of *Nod22 *transcript in common bean treated plants (lower panel). Expression levels of *Nod22 *transcript were determined by qRT-PCR at 2 weeks post inoculation. Mock, mock-inoculated plants; vector, empty vector-inoculated plants; VIGS*Nod22 *P1-P3, *Nod22 *silenced common bean plants. The primers *nod22*-ext., *nod22*-int., and *PvEf1-α *(Table I) were used in this experiment to determine the *Nod22 *transcript abundance. Bars represent the mean value of three biological and technical replicates. Error bars represent standard error. P-values < 0.01.

**Figure 3:**
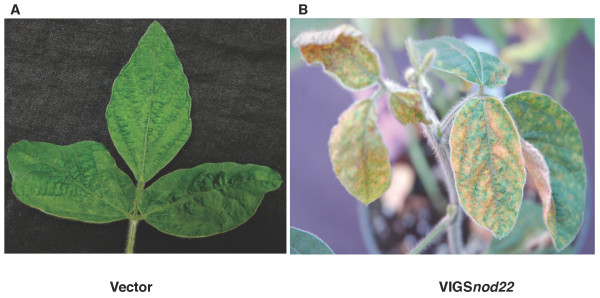
**Virus-induced gene silencing of *GmNod22 *in soybean plants mediated by the BPMV-RNA2 vector**. Silencing of *GmNod22 *induces necrotic phenotypes in 3 week-old soybean plants (VIGS*Nod22*, right panel). Vector control is shown in the left panel.

*PvNod22 *was isolated from a common bean (*Phaseolus vulgaris *L.) cDNA library derived from *Rhizobium*-infected roots [20]. Nod22 deduced sequence reveals regions of high identity to the α-crystallin domain found in α-crystallin lens chaperones and other small heat-shock proteins (sHSPs). Gene sequence comparisons between *PvNod22 *and its homologue in soybean *GmNod22 *(GeneBank accession: CO978845, DB962750, DB979602) revealed an amino acid identity level of 88-89% (See additional file 1: Alignment of Nod22 sequences). The phenotype of *Nod22*-silenced common bean plants was characterized by the development of systemic necrotic lesions and extensive interveinal necrosis, which was apparent two weeks postinoculation (Figure 2, VIGS*Nod22*). A similar phenotype was also observed in *Nod22*-silenced soybean plants (Figure 3, VIGS*Nod22*).

The level of the *Nod22 *mRNA expressed 15 days postinoculation (15 dpi) was determined by using *nod22*-int. and *nod22*-ext. primer sets and the coding sequence of *PvEf1*-α as a reference transcript in common bean (Table 1; see also additional file 2: Annealing positions of *Nod22 *primers). The results indicated that *Nod22 *was not induced during viral infection (Figure 2, mock *versus *vector). Although *Nod22 *expression levels varied in *Nod22*-silenced plants (Figure 2, lower panel; VIGS*Nod22 *P1-P3), these were diminished considerably (up to 50%) compared to mock and vector-treated plants. Similar results were also obtained in *Nod22*-silenced soybean plants (data not shown).

**Figure 4:**
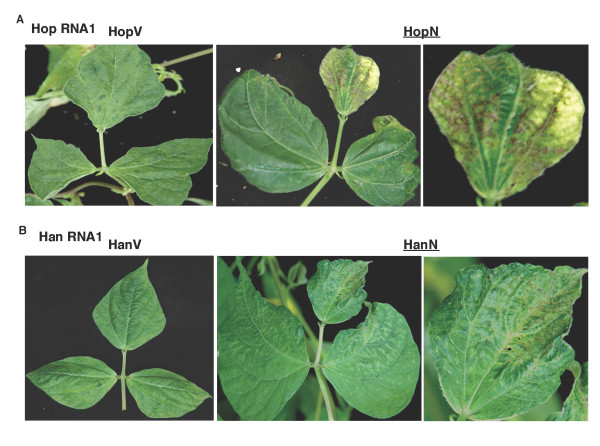
**VIGS phenotype in common bean is independent of the BPMV strain used as a source of RNA1**. (Upper panels). Symptoms and silencing phenotype of common bean plants treated with HopV or HopN. HopV: RNA1, transcript from BPMV strain K-Hop (moderate/relatively severe in soybean) RNA1 and empty vector (V). HopN: RNA1 transcript from BPMV strain K-Hop plus recombinant RNA2-*nod22 *(N). (Lower panels). Same as upper panels except that the RNA1 transcript in the treatments HanV and HanN was derived from the mild strain K-Han. Enlarged views of the middle leaflets are shown in the right panels.

We were also interested in determining whether the source of RNA1 (determinant of symptom severity in BPMV-infected plants [23]) may have a bearing on the resultant silencing phenotype. For this purpose, the transcript of recombinant RNA2 (R2-*Nod22*) was mixed with either RNA1 derived from the mild strain K-Hancock (R1-Han, BPMV-subgroup II [24]) or the relatively severe strain K-Hopkins (R1-Hop, BPMV subgroup I). The treatment involving the R1-Han and R2-*Nod22 *mixture was referred to as HanN. Similarly the treatment involving R1-Hop and R2-Nod22 was designated HopN. The results shown in Figure 4 suggest that, regardless of the differences in virus symptom severity between the two strains, the silencing phenotypes were very similar albeit differing in intensity (Figure 4; compare HopN and HanN).

### Silencing of *SACPDs *in common bean causes similar morphological changes to those induced in soybean plants

In addition to *Nod22*, we silenced *PvSACPDs *in common bean. Expressed sequence tags (ESTs from libraries prepared from pod, nodule or leaf tissues) encoding putative SACPD-like proteins were identified from the database (The DFCI *Phaseolus vulgaris *Gene Index [PhvGI], CV534591, TC14891, TC12707; [25]). Nucleotide sequences of *PvSACPDs *were compared with the soybean genes *GmSACPD-A, B *and *C*. Our analysis indicated that common bean *SACPDs *are related to the soybean isoforms *GmSACPD-A/B *(*PvSACPD-A/B*-like or *A'/B'*) and *C *(*PvSACPD-C*-like or *C'*). Due to the high sequence similarity of *SACPDs *between common bean and soybean, we used a recombinant BPMV-based vector previously used to silence all three *GmSACPDs *in soybean [13]. The recombinant RNA2-*SACPD *construct includes a highly conserved 258 bp fragment from *GmSACPD-A*. This sequence is highly conserved among all three *GmSACPD *isoforms as well as among the *PvSACPDs *isoforms (See additional file 3: Alignment of *SACPD *sequences).

**Figure 5:**
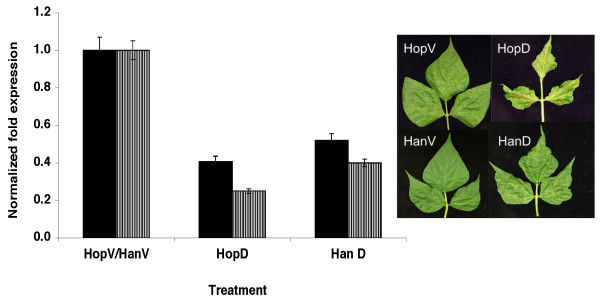
**VIGS of *PvSACPD *in common bean plants by the BPMV-RNA2 vector using two BPMV strains as sources of RNA1**. Variation in *SACPD-C' *transcript relative amounts in common bean plants inoculated with treatments: HopV (RNA1 from strain K-Hop plus empty vector), HanV (RNA1 from strain K-Han plus empty vector), HopD and HanD, RNA1 from strains H-Hop and K-Han, respectively, plus recombinant RNA2-*SACPD *(D). Black solid bars, *SACPD-C' *expression levels in treated plants 15 dpi, stripped bars, *SACPD-C' *expression levels in treated plants 25 dpi. Bars represent the mean value of three biological and technical replicates. Error bars represent standard error. P-values < 0.01. (Right panel). Silencing of common bean *SACPD*s induced a chlorotic/necrotic phenotype, as previously shown in soybean plants [13].

**Figure 6:**
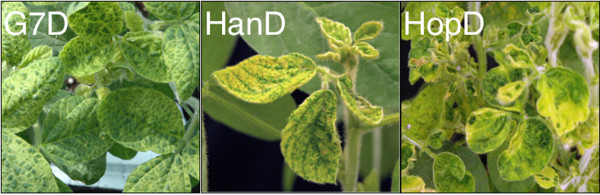
**Silencing phenotypes of *SACPD*s in soybean**. Plants of the soybean cultivar Essex were treated with inocula containing recombinant RNA2-*SACPD *(D) plus RNA1 derived from either strain K-G7 (moderate strain; treatment G7D), strain K-Hop (relatively severe strain; treatment HopD) or strain K-HanD (mild strain; treatment HanD).

The results indicated that *SACPD*-silenced common bean plants indeed contained lower amounts of the *SACPD-C' *transcript 15 and 25 dpi (Figure 5, left panel). Phenotypic silencing was apparent after two weeks and its effect proceeded over time. The phenotypes of plants silenced for *SCAPDs *were similar regardless of the source of RNA1 in the inoculum. As was the case with the *Nod22*-silenced plants, the inoculum containing RNA1 from the mild strain K-Han (R1-Han) and the recombinant RNA2-*SACPD *(D; treatment HanD) produced a silencing phenotype (Figure 5, HanD) similar to, but less intense than, that induced when RNA1 was derived from the moderate/relatively severe strain K-Hop (compare HanD and HopD; Figure 5). Although the VIGS phenotypes of plants treated with HanD and HopD were largely similar, HopD appears to be more efficient in silencing *SACPDs *in common bean (histogram in Figure 5, left panel). This is consistent with the intensity of the phenotypic changes observed in the corresponding *SACPDs*-silenced plants (Figure 5, right panel). It is of interest that, though largely similar, the silencing phenotype induced by HanD in soybean (a treatment including RNA1 from a mild strain) was considerably more intense than that induced by HopD or G-7D (RNA1 from a relatively severe or a moderate strain, respectively) (Figure 6), thus emphasizing that the VIGS phenotypes in both soybean and common bean are independent of the source of BPMV RNA1 (Figures 5 and 6).

### Expression levels of foreign genes from BPMV vectors in common bean

**Figure 7:**
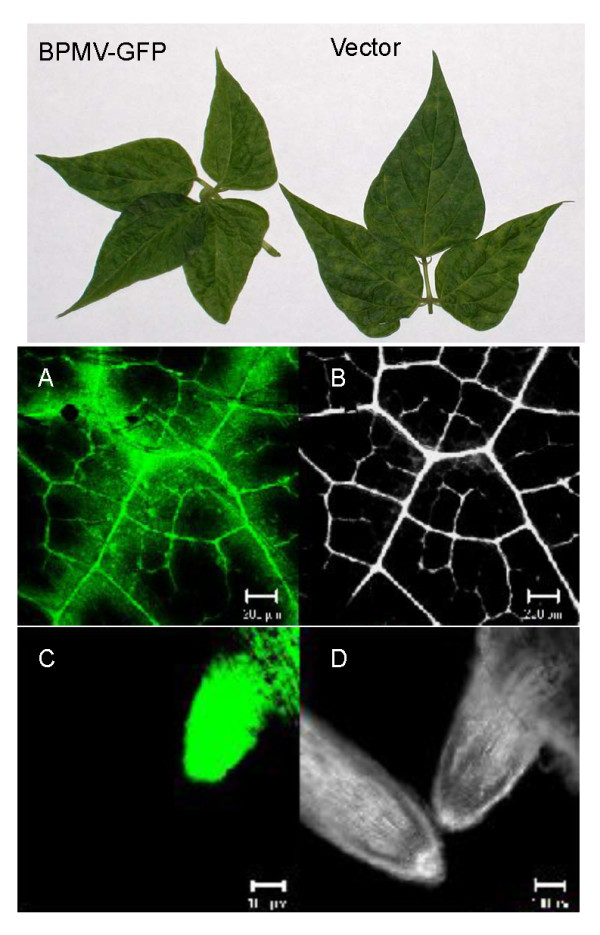
**GFP expression from the BPMV vector in common bean plants**. (Upper panel). Systemic leaves of common bean plants infected with either BPMV-GFP or empty vector showing characteristic chlorotic mottling. (Middle panel). Confocal laser scanning microscopy detection of GFP in systemic leaves. A. Specific fluorescence emitted by GFP in systemic leaves; B. Dark field image. Scale bars are 200 micrometers. (Bottom panel). Confocal laser scanning microscopy detection of GFP in roots of common bean plants. C. Specific fluorescence emitted by GFP in roots of BPMV-GFP (right oriented) or empty vector-treated common bean plants (left oriented); D. Dark field image. Scale bars are 100 μm.

Common bean seedlings were inoculated on their primary leaves with leaf extracts prepared from plants infected with empty vector and the recombinant vector carrying GFP sequence (BPMV-GFP). The first and second trifoliolate leaves as well as the roots of GFP-expressing plants, but not the empty vector-treated plants showed intense fluorescence under UV light 15 dpi. Confocal microscopy further confirmed the expression of GFP in the tissues of BPMV-GFP-treated common bean plants (Figure 7). GFP expression was intense and stable through at least four serial passages.

## Discussion

We used the BPMV-based vector, previously described for applications in soybean [12], for gene silencing studies in common bean. To our knowledge, this is the first report that documents efficient and stable silencing of endogenous genes in common bean by VIGS. We also demonstrated stable and efficient systemic expression of the marker protein GFP, thus establishing that the BPMV vector is suitable for expression of heterologous proteins in common bean. The BPMV-GFP vector was previously shown to be stable after four serial passages in soybean cv Essex [12] and three passages in common bean cv. Black Valentine [[12]; reported as data not shown]. Here we extend this finding by using confocal microscopy to demonstrate efficient GFP expression in all common bean tissues where BPMV is detected.

The BPMV-RNA2 vector is the only available plant-virus-based vector that is appropriate for stable expression of foreign proteins in common bean. Although the cowpea mosaic virus (CPMV)-RNA2 vector [26] could potentially be used for these applications, there is concern about its genetic stability due to homologous recombination as a consequence of the duplication of the cleavage sites. In engineering the BPMV-RNA2 vector, we altered the nucleotide sequence of the duplicated regions without affecting its amino acid sequence in order to minimize the chances of homologous recombination [12]. Recently, Zhang et al [27] reported the development of a modified BPMV-RNA2 expression vector similar to that previously reported for the CPMV-RNA2 vector [26], in which they replaced the duplicated Q/M cleavage site with a foot and mouth virus autocatalytic 2A peptide. Incorporation of a 2A sequence between GFP (or protein of interest) and L-CP precludes the need to duplicate viral sequences and therefore is advantageous in terms of the genetic stability of constructs [26,28]. Cleavage at a 2A site occurs between the last two amino acids of the sequence, resulting in that GFP (or the protein of interest) having all but one of the 2A amino acids attached to its C terminus, whereas the L-CP has only an additional proline at its N-terminus. Thus, the expressed protein (GFP) using such a vector is still not authentic in that it contains ~20 or more amino acids derived from the 2A sequence. Furthermore, due to the incompleteness of the cleavage mediated by 2A peptide, a significant portion of the L-CP subunits could carry GFP fused to their N termini, and these fusion proteins can be incorporated into virus particles. Such particles are predicted to be empty (noninfectious) since the N-terminus of the L-CP, now fused to GFP, is not available to interact with the viral RNA [28]. Because of these reasons, the stability of the recombinant vector could be difficult to maintain through serial passage, a matter that was not tested by Zhang et al. [27].

In addition to protein expression in common bean, we used the BPMV-RNA2 vector to demonstrate efficient and stable silencing of endogenous genes coding for Nod22 and SACPDs. Nod22 is a small-heat shock protein (sHSP). sHSPs constitute a diverse family of molecular chaperones, crucial components of plant stress tolerance and normal development. Indeed, *Nod22 *transcripts are accumulated in response to pathogens [29] and root nodule development [20]. Remarkably, when this protein is over-expressed in *E. coli*, it confers protection against oxidative stress [20]. Phenotypic results on functional analysis of Nod22 in common bean and soybean, achieved in this work, characterized by the appearance of systemic necrotic lesions and interveinal necrosis (Figure 2) further supported the hypothesis that Nod22 is involved in plant defense responses.

Likewise SACPDs are also well known to be involved in plant defense [30]. SACPD is the archetype member of a family of soluble fatty acid (FA) desaturases; these enzymes play an important role in regulating the overall levels of desaturated FAs in the cell. A mutation in the SACPD encoding *SSI2 *gene in Arabidopsis results in constitutive expression of pathogenesis-related (*PR*) genes, spontaneous lesion formation, increased accumulation of the phytohormone salicylic acid, and enhanced resistance to bacterial and oomycete pathogens [31]. Analysis of suppressor mutations has shown that the altered defense signaling in the *ssi2 *plants is a result of their altered FA profile [32-35]. Silencing *SACPD *genes in soybean and rice also induce similar phenotypes as in Arabidopsis [13,36]. Our work shows that this FA-derived defense-signaling pathway is also conserved in common bean (Figure 5).

The choice of BPMV strain(s) to use for VIGS studies in common bean is not an issue since the symptoms induced by infection involving RNA1s from both strain K-Han (mild symptoms on soybean) and K-Hop (moderate/relatively severe symptoms on soybean) are relatively mild on common bean and do not appear to interfere with the VIGS phenotypes (Figures 4 and 5). Provided efficient silencing can be achieved, it is obviously preferable to use symptomless or very mild virus strains in VIGS studies, mainly because of easier detection of silencing phenotypes. However, to use such symptomless virus strains, the inoculation protocol becomes cumbersome since the inoculum has to be increased in an alternative symptomatic host. For example, ALSV inoculum has first to be increased in *Chenopdium quinoa *and following two passages in *C. quinoa*, total RNA was prepared from the infected leaves and used to inoculate soybean leaves via particle bombardment [19]. Furthermore, It should be emphasized that the virus-induced symptoms become evident long after the onset of virus infection and replication. Using genomics and proteomics technologies it was possible to identify at least 250 host factors that affect virus replication [37]. Thus it is much more complicated than perceived in previous reports that commented on the rationale for using symptomless virus strains for VIGS work [17,19,27,38].

Agroinfection is the most cost effective and efficient method for inoculating plants with virus-based vectors [39] and thus ideal for high-throughput applications including screening of cDNA libraries or EST collections. Although agroinfection has been used successfully for several different plant species, it is not yet amenable for use in common bean or soybean. We routinely and successfully use the transcript rub-inoculation method to introduce the BPMV vector into soybean and common bean. The efficiency of the transcript inoculation method depends on the quantity and quality of transcripts, regardless of the source of RNA1. In our experience, we obtain better RNA1 transcript yield with plasmid pGHoR1 than with plasmids pGG7R1 or pCRHaR1 [23]. Considering that the VIGS phenotype is independent of the source strain of RNA 1, we routinely use pGHoR1 in our studies. We generally obtain 40-80% infection rate (average 60%), and this is very satisfactory for our purpose since we get 100% infection with first passage plants. Use of biolistics to introduce the vector into individual plants with 35S promoter-driven DNA constructs using a gene gun [27,38] seems unnecessary considering the stability of our vector and the need to use transcripts only once for inoculation. We and others have successfully silenced or over-expressed a large number of soybean and common bean genes using the transcript rub-inoculation method [[13-16,40] and Ghabrial, unpublished]. Until *Agrobacterium*-mediated inoculation methods become available for soybean and common bean, the transcript rub inoculation method has proven efficient for our laboratory.

## Conclusion

The development of BPMV-VIGS vector is an important advance for the common bean research community since rapid functional studies in common bean have been greatly restrained due to the lack of appropriate tools. In this work, we have used the BPMV-based vector, previously described for applications in soybean [12], for gene silencing studies in common bean. As far as we know this is the first report that documents efficient silencing of endogenous genes in common bean by VIGS. Furthermore, we demonstrated efficient systemic expression of the marker protein GFP thus establishing that the BPMV vector is suitable for expression of heterologous proteins in common bean.

## Competing interests

The authors declare that they have no competing interests.

## Authors' contributions

CDC, PA and SAG designed the experiments; CDC and PA performed the experiments; CDC, FS, AK and SAG analyzed the data, CDC and SAG wrote the paper with contributions from all the authors. All authors read and approved the final manuscript.

## Supplementary Material

Additional file 1 **Alignment of *Nod22 *sequences**. Partial alignment of nucleotide sequence of *PvNod22 *with its homolog from soybean *GmNod22*. The alignment includes the region of *PvNod22 *used for generating the *Nod22 *silencing fragment. Numbers indicate nucleotide positions. Nucleotide sequences of *PvNod22 *[20] and *GmNod22 *(GeneBank accession numbers: CO978845, DB962750, DB979602) are 90% identical within this region. Nucleotide positions that are different between the two sequences are shaded. Click here for file

Additional file 2 **Annealing positions of *Nod22 *primers**. Full-length nucleotide sequence of *Nod22 *cDNA is shown with the 5' UTR underlined and the sequence corresponding to the silencing fragment shaded. The initiation (ATG) and termination (GTA) codons are printed in bold. The nucleotide sequences of the primer pairs *nod22*-ext, *nod22*-int. and *nod22 *are indicated by solid red, solid blue and dashed arrows, respectively. Click here for file

Additional file 3 **Alignment of *SACPD *sequences**. Nucleotide sequence alignment of the region corresponding to the *GmSACPD *silencing fragment in three soybean SACPD-coding genes (*GmSACPD*-*A, B*, or *C*) and common bean SACPD genes (*PvSACPD*-*A'/B' *and *C*'). Numbers indicate nucleotide positions. The nucleotide sequence of the *GmSACPD *fragment used to induce *SACPD *silencing in soybean and common bean is underlined. The percent identity between the silencing fragment and *PvSACPD-A'/B' *is 93% while the percent identity between this fragment and *C*' is 72%. Conserved nucleotides between the silencing fragment and the different *SACPD sequences *are shaded Click here for file
