# Supplementary material for: An effective virus-based gene silencing method for functional genomics studies in common bean
Source: Plant Methods. 2011 Jun 13;7:16. doi: 10.1186/1746-4811-7-16 (PMC3141803; doi:10.1186/1746-4811-7-16)
Supplement: Additional file 1 — Alignment of Nod22 sequences. Partial alignment of nucleotide sequence of PvNod22 with its homolog from soybean GmNod22. The alignment includes the region of PvNod22 used for generating the Nod22 silencing fragment. Numbers indicate nucleotide positions. Nucleotide sequences of PvNod22 [20] and GmNod22 (GeneBank accession numbers: CO978845, DB962750, DB979602) are 90% identical within this region. Nucleotide positions that are different between the two sequences are shaded. [file 1746-4811-7-16-S1.PDF]

Pvnod22 151 CAGGCGCTGTTGGGAATCGCTGGCAGCAAGAAGCTCCGGCGACT

Gmnod22 CAGGCGCTGTTGGGGATCGCCGGGA---AGAAGCTCCGGCGACT

Pvnod22 195 CCCGCACGTGTTCAGCTGCGTCCTGGAGCTCCCGTTCCGCTCCG

Gmnod22 ACCGCACGTGTTCAGCTGCGTGCTGGAGCTGCCCTTCCGCTCCG

Pvnod22 239 ACGCCGACGTGGTGGTGGAGGAGGACCCCGACTGCTTCCGCTTC

Gmnod22 ACGCCGACGTTGCGGTGGAGGAAGCCCCCGACTGCTTCCGCTTT

Pvnod22 283 GTGGCGGAGACTGAGGGTATCGGCGACGTGAGGGCGCACACGAT

Gmnod22 GTGGCGGAGACCGATGGAATCGGCGACGTGAGGGCGCACACGGT

Pvnod22 327 CGAAATCCACCCCGGCGTGACGAAGATCGTGGTGAGGGACGGCG

Gmnod22 GGAAATCCACCCTGGCGTGACGAAGATCGTGGTGAGGGACGGCG

Pvnod22 371 GTTCGGTGGAGCTCTCGCTCGACCAGCTCGAACTGGATATGTGG

Gmnod22 GCTCGGTGGAGCTCTCGCTCGACCAGCTGGAGCTCGATATGTGG

Pvnod22 415 AGGTTCCGTTTACCAGAATCGACGCGGCCGGAGCTCGCGAGCGC

Gmnod22 AGGTTCCGGTTGCCGGAATCGACTCGGCCGGAGCTCGCCAGCGC

Pvnod22 459 GGTGTTCGTAGACGGCGAGCTCATCGTGACGGTGCCGAAGGGGC

Gmnod22 GGTGTTCGTCGACGGCGAGCTCATCGTCACGGTGCCGAAGGGGC

Pvnod22 503 ACGGAGAGG 512

Gmnod22 ACGAAGAGG

Additional file 1
