# Supplementary material for: An effective virus-based gene silencing method for functional genomics studies in common bean
Source: Plant Methods. 2011 Jun 13;7:16. doi: 10.1186/1746-4811-7-16 (PMC3141803; doi:10.1186/1746-4811-7-16)
Supplement: Additional file 2 — Annealing positions of Nod22 primers. Full-length nucleotide sequence of Nod22 cDNA is shown with the 5' UTR underlined and the sequence corresponding to the silencing fragment shaded. The initiation (ATG) and termination (GTA) codons are printed in bold. The nucleotide sequences of the primer pairs nod22-ext, nod22-int. and nod22 are indicated by solid red, solid blue and dashed arrows, respectively. [file 1746-4811-7-16-S2.PDF]

ACGCACATATTCCGCATCTATTCTTCTCTTCTGCTCACACATTTCATCAATTCGTCGCAGCATCTCAATCT

CCCAACATTTTCTCCACAAAGGGAAAATAAATCTTAATAACAATAAAATAAAAAGGGAATCACGTCTTTCC

AATCCGCCAAACGCGATCGTCCCGGCCGCAAATTGAAATTAGGGTTAGGGTTTTCATCGTCTTCGCCCGAA

AAAGAAAGGGGGAAGTGGGAAGGAAGACGCGAACAACCGTTCCTTT**ATG**ATACAAAAGGTGTCTCTGATCT

TCATCGTCATCATCGCTGTATTAATCTTGGTGTTGCAATCCTCTGCGGCGTTCCACAGTAGCAAAAACAGC

ATCATGAAGGTCCACCCTGTTCCGCGAAAACGCAACATCTCAATCCAATTCGGCGTCGACGGGGGGAATCC

CATGTCGGAGGCGCAGGCGCTGTTGGGAATCGCTGGCAGCAAGAAGCTCCGGCGACTCCCGCACGTGTTCA

GCTGCGTCCTGGAGCTCCCGTTCCGCTCCGACGCCGACGTGGTGGTGGAGGAGGACCCCGACTGCTTCCGC

TTCGTGGCGGAGACTGAGGGTATCGGCGACGTGAGGGCGCACACGATCGAAATCCACCCCGGCGTGACGAA

GATCGTGGTGAGGGACGGCGGTTCGGTGGAGCTCTCGCTCGACCAGCTCGAACTGGATATGTGGAGGTTCC

GTTTACCAGAATCGACGCGGCCGGAGCTCGCGAGCGCGGTGTTCGTAGACGGCGAGCTCATCGTGACGGTG

CCGAAGGGGCACGGAGAGGAAGACGGGGATGGTGATAGGGTTATGGGTGGTGGTAGACTTGTGCTTGTACA

G**TGA**

Additional file 2
