# Supplementary material for: An effective virus-based gene silencing method for functional genomics studies in common bean
Source: Plant Methods. 2011 Jun 13;7:16. doi: 10.1186/1746-4811-7-16 (PMC3141803; doi:10.1186/1746-4811-7-16)
Supplement: Additional file 3 — Alignment of SACPD sequences. Nucleotide sequence alignment of the region corresponding to the GmSACPD silencing fragment in three soybean SACPD-coding genes (GmSACPD-A, B, or C) and common bean SACPD genes (PvSACPD-A'/B' and C'). Numbers indicate nucleotide positions. The nucleotide sequence of the GmSACPD fragment used to induce SACPD silencing in soybean and common bean is underlined. The percent identity between the silencing fragment and PvSACPD-A'/B' is 93% while the percent identity between this fragment and C' is 72%. Conserved nucleotides between the silencing fragment and the different SACPD sequences are shaded [file 1746-4811-7-16-S3.PDF]

```
GmSACPD–A 330   GAGCTTCCAGATGATTACTTTGTTGTTCTGGTCGGAGACATGATCACAGAGGAAGC
GmSACPD–B 330   GAGCTTCCAGATGATTACTTTGTTGTTCTGGTCGGAGACATGATCACAGAGGAAGC
PvSACPD–A′/B    GAGCTTCCAGATGATTACTTTGTTGTTCTGGTTGGAGACATGATCACAGAGGAAGC
GmSACPD–C 306   GAGTTACCTGATGAGTACTTTGTGGTGCTGGTGGGTGATATGGTCACCGAGGACGC
PvSACPD–C′      GAACTACCTGACGAGTACTTCGTGGTGCTGGTGGGTGACATGGTCACCGAAGACGC

GmSACPD–A       CCTGCCTACTTACCAAACAATGTTAAATACTTTGGATGGAGTTCGTGATGAAACAG
GmSACPD–B       CCTGCCTACTTACCAAACAATGTTAAATACTTTGGATGGAGTTCGTGATGAAACAG
PvSACPD–A′/B′   CCTACTTACCAAACAATGTTAAATACATTGGATGGAGTTCGTGATGAAACAGGCGC
GmSACPD–C       CCCACTTACCAGACCATGATCAACAACCTTGATGGAGTGAAAGATGACAGCGGCAC
PvSACPD–C′      CCCACCTACCAGACTATGATCAACAACCTCGACGGGCTGAGAGACGAGTGCGGAAC

GmSACPD–A       CTTACTTCCTGGGCAATTTGGACAAGGGCATGGACTGCTGAAGAAAACAGACACGG
GmSACPD–B       CTTACTTCCTGGGCAATTTGGACAAGGGCATGGACTGCTGAAGAAAACAGACACGG
PvSACPD–A/B′    CTTACTTCCTGGGCAGTCTGGACAAGGGCATGGACTGCTGAAGAAAATCGACATGG
GmSACPD–C       CCGAGCCCGTGGGCCGTGTGGACCCGGGCCTGGACCGCCGAGGAAAACAGACACGG
PvSACPD–C′      CCAAGCCCGTGGGCTGTGTGGACCCGGGCCTGGACGGCAGAAGAAAACAGACACGG

GmSACPD–A       TGATCTTCTTAACAAATATCTTTACTTGAGTGGTCGAGTTGACATGAAACAAATTG
GmSACPD–B       TGATCTTCTTAACAAATATCTTTACTTGAGTGGTCGAGTTGACATGAAACAAATTG
PvSACPD–A′/B′   TGATCTTCTTAACAAGTATCTTTACTTTAGTGGACGAGTTGACATGAGACAAATTG
GmSACPD–C       GGATCCTGCTCAGAACTTATTTGTATCTCTCTGGGAGGGTTGACATGGCTAAGGTC
PvSACPD–C′      TGATCTTGCTCAGAACTTACTTGTACCTCTCTGGTCGCGTTGACATGGCTAAGGTT

GmSACPD–A       AAAAGACAATTCAGTACCTTATTGGG   606
GmSACPD–B       AAAAGACAATTCAGTACCTTATTGGG   606
PvSACPD–A′/B′   AGAAGACAATTCAGCACCCGGATGGG
GmSACPD–C       AAAAGACCGTACATTACCTCATTTCA   582
PvSACPD–C′      AAAACTTTGTCCATTACCTCATCGGA
```

Additional file 3
